# Not Your Typical Tonsil: Metastatic Merkel Cell Carcinoma or Primary Disease?

**DOI:** 10.7759/cureus.14604

**Published:** 2021-04-21

**Authors:** Jason C DeGiovanni, Cathleen C Kuo, Ellen L Tokarz, Ameer T Shah, Miriam OLeary

**Affiliations:** 1 Otolaryngology - Head and Neck Surgery, University at Buffalo-State University of New York, Buffalo, USA; 2 Otolaryngology - Head and Neck Surgery, Tufts Medical Center, Boston, USA

**Keywords:** merkel cell carcinoma, merkel cell carcinoma of unknown primary, palatine tonsil

## Abstract

Merkel cell carcinoma (MCC) is a rare, aggressive neuroendocrine tumor that almost always presents as a cutaneous lesion in the sun-exposed areas on the bodies of elderly white males. Metastasis to lymph nodes in the presence or absence of a known primary site and occurrence of these tumors in non-sun-exposed sites have also been described; however, an incidence of recurrent disease arising in the palatine tonsil in the absence of any detectable primary lesion has never been reported in the literature. In this report, we discuss a case of a 72-year-old female who was found to have a single axillary lymph node, which was resected and proved to be positive forMCC of unknown primary (MCCUP). Since there was no evidence of additional disease, the patient elected not to pursue adjuvant therapies. Six and a half months later, she presented with a complaint of dysphagia and a right-sided exophytic tonsillar mass. Tonsillectomy revealed MCC with no detectable primary cutaneous lesion. She received adjuvant therapy with avelumab and demonstrated a complete response after one year of bi-weekly treatments. Seven months following cessation of adjuvant treatments, surveillance positron emission tomography (PET) revealed enlarged retroperitoneal, pretracheal, periaortic, and left axillary lymph nodes concerning for recurrence. She elected to forgo additional biopsies and restarted avelumab the following month. She continues to be followed up on a monthly basis.

## Introduction

Merkel cell carcinoma (MCC) is widely regarded as an aggressive malignant neuroendocrine tumor that is thought to arise from the Merkel cell, a specialized neuroepithelial cell that may have light touch receptor function [1]. It is most commonly seen in sun-exposed areas on the bodies of elderlywhite males, such as the face and extremities, and it usually presents as a nodular erythematous or violaceous lesion [2-3]. MCC accounts for only 0.8% of all cutaneous skin cancers with an annual incidence of approximately 0.6 per 100,0000 patients [2]. However, its incidence was noted to increase four-fold between 1986 and 2006, which was largely attributed to improved detection methods. The condition has a mortality rate of 46%, which makes the diagnosis of this pathologyconcerning [2,4-5]. Although the exact pathophysiology of the disease remains unknown, frequently reported risk factors are ultraviolet radiation exposure and immunosuppression. Interestingly, over one-third of patients with clinically detectable lymph nodes at diagnosis do not have an identifiable skin lesion [2,6]. This group of patients is considered to have nodal MCC of unknown primary (MCCUP). With the increasing incidence of this entity, the clinical management of MCCUP patients has been found challenging because they paradoxically tend to have a more favorable prognosis than patients with a known primary. The largest review of patients with nodal MCCUP reported since 1988 found the distribution of nodal involvement to be 42.3%, 17.7%, and 40% for the cervical, axillary, and inguinal nodes respectively [7]. There is no established treatment guideline for MCCUP; however, surgical excision followed by adjunctive chemoradiation therapy is the most commonly used modality. More recently, immunotherapies with agents such as avelumab have been approved for treatmentas well.

The purpose of this report is to discuss a rare case of a recurrent MCCUP arising in the palatine tonsil of an elderly woman. We also reviewfour other reported cases of MCC of the palatine tonsiland engage in a brief discussion highlighting the current research aimed at understanding MCCUP as its own potential disease entity.

## Case presentation

**Figure 1:**
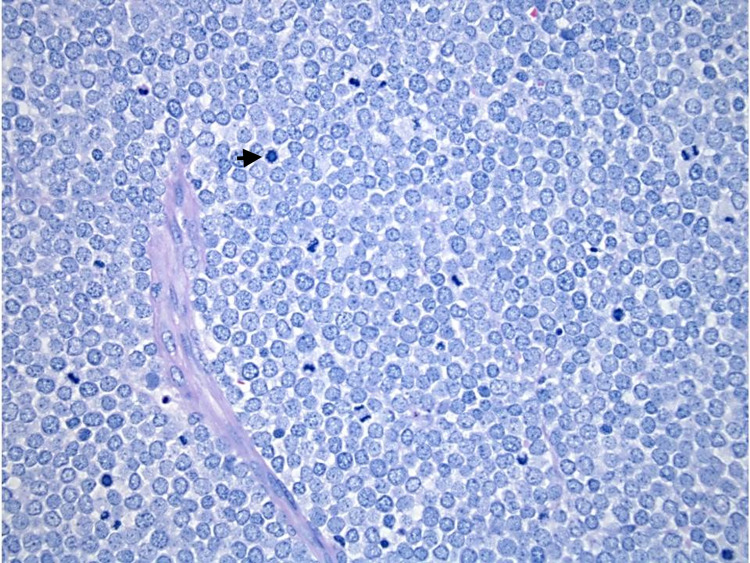
Axillary lymph node at 40x magnification Hematoxylin and eosin stain of right axillary lymph node at 40x magnification. Microscopically, MCC is composed of sheetsof monotonous cells with scant pale cytoplasm and round vesicular nuclei with finely speckled chromatin (arrow) MCC:Merkel cell carcinoma

**Figure 2:**
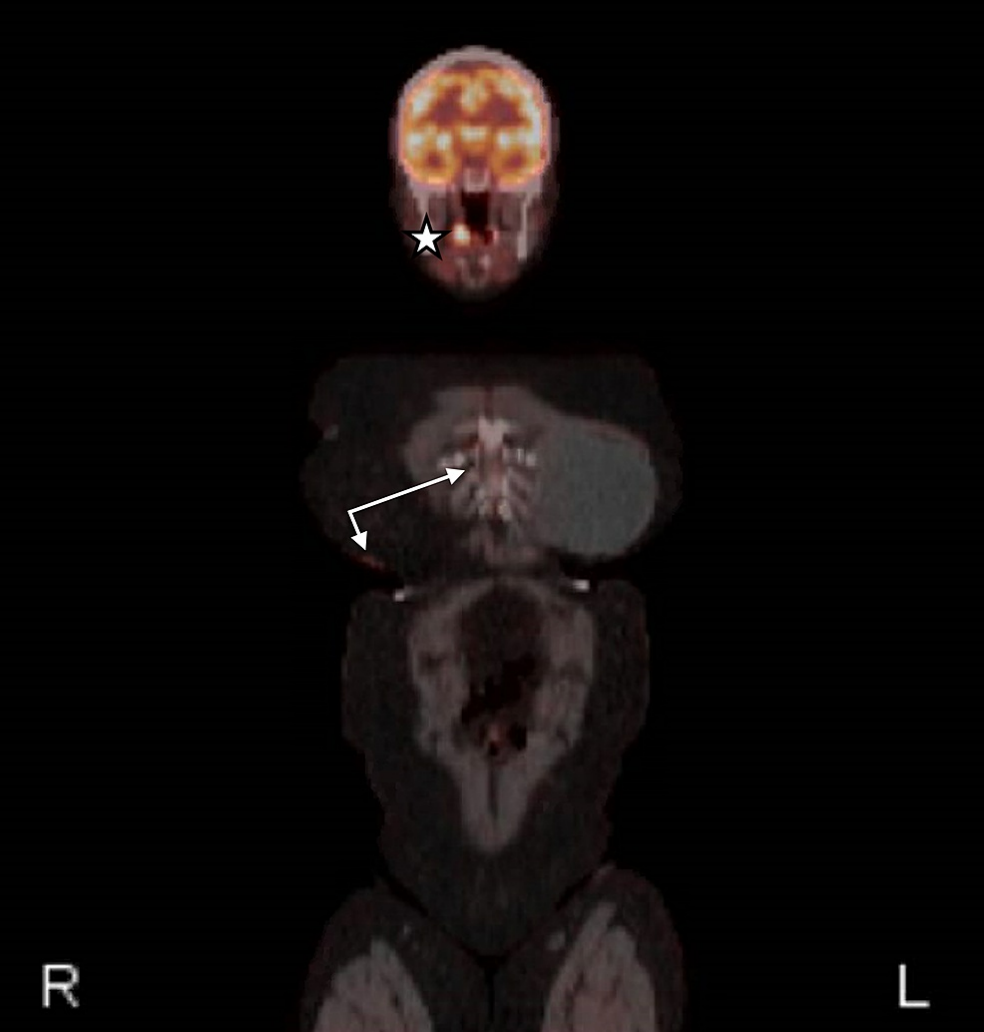
PET scan six months following axillary node dissection Surveillance PET scan performed six months after the patient's right axillary node dissection demonstrating interval appearance of mediastinal, hilar, and porta hepatis lymphadenopathy (arrows). Additionally, intense uptake in the right palatine tonsil was noted (star) PET: positron emission tomography

A 72-year-old woman with a history of left breast cancer treated with total mastectomy was found to have a left axillary node measuring 7 cm on routine screening mammography and ultrasound five years after undergoing the treatment. Core needle biopsy of this node demonstrated poorly differentiated neuroendocrine carcinoma that, after thorough pathologic and immunohistological examination, proved to be consistent with MCC (Figure 1). Imaging studies [positron emission tomography (PET) and non-contrast MRI] and thorough dermatologic examinations over the subsequent month failed to reveal any evidence of a primary lesion. The patient underwent treatment with a right axillary dissection and removal of 14 lymph nodes; only a single node was positive for MCC. A postoperative MRI study with contrast demonstrated no signs of residual axillary nodal disease. The patient decided to forgo any adjunctive local radiotherapy (RT). She was evaluated again by her dermatologist in the following months and still had no cutaneous lesions. Six months following the axillary lymph node dissection, a PET scan demonstrated interval development of supraclavicular, mediastinal, hilar, and porta hepatis lymphadenopathy. Additionally, intense uptake in the right oropharyngeal region was noted (Figure 2). Biopsy of her mediastinal node showed acid-fast bacilli (AFB)-negative granulomatous changes and was negative for MCC. Given the oropharyngeal findings on imaging and the recent appearance of a white spot on her right tonsil, she was referred to our service for further evaluation.

**Figure 3:**
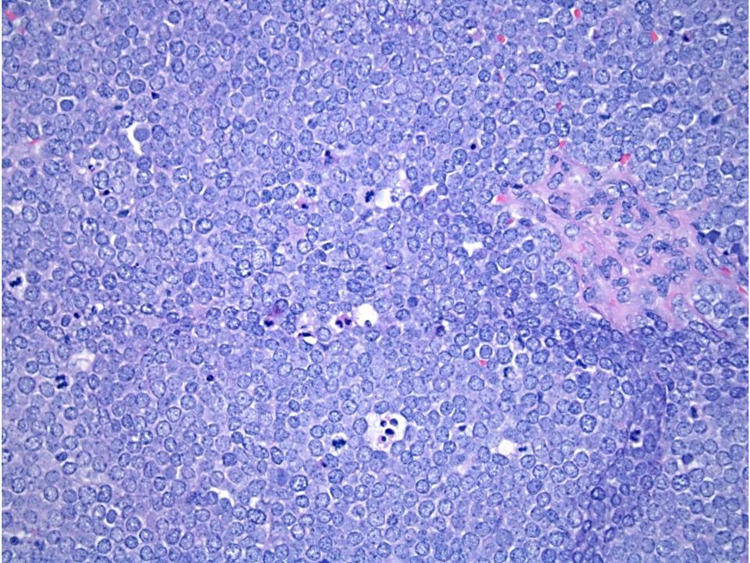
Right palatine tonsil at 40x magnification Hematoxylin and eosin stain of right palatine tonsil at 40x magnification.Illustratedagainis the characteristic pattern of monotonous cells with scant pale cytoplasm and round vesicular nuclei with finely speckled chromatin

A review of a CT scan demonstrated a mass that appeared to emanate from the right lateral oropharyngeal wall correlating with the area of fluorodeoxyglucose(FDG) avidity on her most recent PET scan. During her initial visit with our team, she described a three-week history of throat pain, which coincided with the appearance of her tonsillar lesion. She also noted associated dysphagia, hoarseness, and mild right otalgia but denied any weight loss or decrease in her oral intake. Her exam was notable for an exophytic, friable-appearing mass in the region of her right tonsil. The patient underwent a right tonsillectomy, and pathology was consistent with MCC with extensive surface colonization by *Actinomyces* (Figure 3). Subsequently, the patient agreed to begin a one-year course of adjuvant therapy with avelumab 10 mg/kg every two weeks. Two months later, after five cycles of treatment, a PET scan noted an interval decrease in the size of suspected pathological lymphadenopathy. After 11 cycles of treatment, another PET scan revealed complete resolution of her lymphadenopathy and no evidence of disease. Repeat scans at the completion of her one-year treatment continued to show no evidence of disease. She was scheduled for a surveillance PET scan three months after treatment completion, but it was not performed due to an insurance issue. Unfortunately, a PET scan performed six months after treatment completion revealed a new interval enlargement of multiple retroperitoneal, pretracheal, and left axillary lymph nodes. Repeat imaging the following month confirmed persistent, and increasing, lymphadenopathy in these regions and new uptake in a left upper periaortic node. After much discussion, the patient elected to forgo any additional biopsies and agreed to resume adjuvant therapy with avelumab. The patient is currently three years from the initial diagnosis of axillary lymph node MCCUP and 26 months from pathologic diagnosis of tonsillar MCC and is without any pathological evidence of disease progression. She continues with her bi-weekly avelumab treatments and is closely monitored for disease progression with regular follow-ups.

## Discussion

MCC of the skin is a rare entity, and lymph node disease in the absence of a known primary site is exceedingly rare. Microscopically, MCC is a diffusely infiltrative neoplasm, although trabecular growth may also be seen. It is composed of sheets of monotonous cells with scant pale cytoplasm and round vesicular nuclei with finely speckled chromatin. Multiple nucleoli are often present and mitotic activity is brisk [1,3].The neuroendocrine nature of this tumor can be confirmed with immunohistochemical stains for synaptophysin and chromogranin A, as well as neuron-specific enolase (NSE). In addition, the tumor cells also show positivity for low molecular weight keratins, notably cytokeratin 20 (CK20), with staining in a perinuclear dot-like fashion [4]. To the best of our knowledge, only four other cases of MCC of the palatine tonsil have been reported in the literature. Of these cases, only one patient had no identifiable primary site or known history of MCC.

Of the previously reported cases of MCC occurring in the palatine tonsil, only one case was noted in a patient without a primary site or known history of MCC. Jinkala et al. reported the unusual case of an 80-year-old man who presented with a one-month history of throat pain and was found to have a right tonsillar mass and an enlarged ipsilateral right level II cervical lymph node. Excision of the tonsillar mass confirmed MCC in the tonsil but only lymphoid hyperplasia was noted in fine-needle biopsy of the enlarged lymph node. No additional treatment or follow-up information was provided [8]. Similar to our case, the patient had no biopsy-proven MCC in what were considered enlarged pathologic nodes on imaging. In our case, the patient had supraclavicular, mediastinal, hilar, and left axillary node enlargement; however, analysis of a mediastinal node failed to reveal MCC. The absence of disease in the mediastinal nodes certainly does not preclude its existence in the other nodal groups but makes it much less likely.

In the four other cases of MCC affecting the palatine tonsil, all patients had a history of treatment for known cutaneous MCC. Tesei et al. reported the first case of MCC occurring in the left palatine tonsil of a 69-year-old male. This patient presented with an 18-month history of progressive odynophagia after the resection of acutaneous MCC on his left forearm for which he had also received adjuvant RT. After the tonsillectomy, the patient was referred for palliative chemotherapy and no additional follow-up information was provided by the authors [9]. Similarly, Miyagishima et al. reported the case of a 68-year-old man with a history of cutaneous MCC of the left forearm three years prior, who presented with bilateral cervical, left axillary, and left supraclavicular adenopathy, as well as an ulcerating lesion of his right palatine. He underwent right tonsillectomy and received adjuvant chemotherapy with cyclophosphamide, doxorubicin, and vincristine and was noted to be disease-free after three cycles of treatment. Three months following the cessation of adjuvant treatment, he was disease-free and no additional follow-up data was provided by the authors [10]. Similar to the previously described case, this patients initial cutaneous lesion was also treated with adjuvant RT. In contrast to the present case, these two cases of MCC in the palatine tonsil occurred in patients with widely metastatic disease in the context of a known cutaneous primary. Vasileiadis et al. reported the third case in a 61-year-old male who presented with a tonsillar mass three years after undergoing treatment for cutaneous MCC on his left shoulder with excision and radiation. Their patient presented with a seven-week history of moderate dysphagia, associated foreign body sensation, and snoring. The patient had a total tonsillectomy and potassium titanyl phosphate(KTP) laser-assisted palatoplasty followed by RT to bilateral necks with concomitant chemotherapy. At the nine-monthfollow-up, there was no recurrent disease reported [11].

Lastly, Nagy et al. reported a case of MCC in the left palatine tonsil of an 86-year-old female. Notably, this patient had a history of MCC of her upper lip, which had been treated with cryosurgery seven years earlier. The patient underwent complete resection of the tonsil but refused adjuvant therapy options and died one month later at the age of 86 years.Their report is interesting in that it is the only other reported occurrence in a female and at the time the patient presented with the tonsillar mass, there was no nodal disease present. The authors also investigated the relationship between her primary cutaneous lesion and presumed recurrent tonsillar lesion with molecular analysis. Analysis of the two samples with comparative genomic hybridization revealed different gene copy numbers at 31 chromosomal locations, which led to the conclusion that the recurrence represented a second field lesion, rather than a metastatic or separate primary lesion [12].

Given that between one-third and one-half of all patients with MCC do not present with a primary cutaneous lesion, efforts to elucidate whether MCCUP represents its own disease entity have been made. These efforts were prompted mainly by the fact that patients with MCCUP have been observed to have significantly better survival rates compared to those with MCC of known primary (MCCKP) with the same stage of disease [7]. Recently, Vandeven et al. conducted a retrospective analysis of 123 patients with or without a known primary cutaneous lesion and investigated markers of immunity by looking at Merkel cell polyomavirus (MCPyV) antibody titers, and tumor mutation burden by determining the number of somatic nonsynonymous mutations indicative of established ultraviolet-induced mutation signatures implicated in MCC and survival rates between the two groups. In their study, they divided patients with MCCUP and MCCKP into two stages: IIIB and IV [6]. Stage IIIB patients presented with the disease in skin draining lymph nodes such as the parotid, axillary, or inguinal nodal groups, while stage IV patients presented with the disease in non-skin draining nodes such as mediastinal, periaortic, or retroperitoneal nodal groups. The authors found that patients with MCCUP stage IIIB and IV had significantly higher disease-specific survival, overall survival, and recurrence-free survival compared to patients with MCCKP in both stages. No differences in MCPyV status were observed between the groups; however, patients with MCCUP were found to have significantly higher MCPyV oncoprotein antibody titers than patients with MCCKP, which led the authors to consider that as a potential reason for the survival advantage. Additionally, patients with MCCUP were noted to have a higher tumor mutational burden compared to those with MCCKP, leading the authors to conclude that patients with MCCUP have tumors with increased neoantigen presentation and immunogenicity, which they hypothesized may also account for survival differences. In the context of the present case, these results are difficult to interpret given that our patient initially presented with the biopsy-proven disease in a skin draining node and later developed the disease in non-draining lymphoid tissue, namely, the palatine tonsil. Thus, while the study by Vandeven et al. does point to some potentially important differences between MCCUP and MCCKP, our case certainly highlights the need for further study and clarification.

With the exception of the case described by Jinkala et al., one similarity among all these casesis the relatively long period between their initial diagnosis and presentation for recurrence. Metastatic lesions, especially those with a well-established history of being aggressive, would not have long latency periods. Coupled with the data from Nagy et al.s report, the long time until recurrence strongly suggests that the cases of palatine tonsillar MCC that have been reported are unlikely to represent the metastatic disease in the instances where the disease was preceded by cutaneous disease. It is noted that the seven months until disease recurrence seen in our patient does contrast with other reported cases with longer time intervals between occurrences.

## Conclusions

MCC of the palatine tonsil is extremely rare, with this case representing only the fifth reported occurrence of the condition. Additionally, all prior cases occurring in the palatine tonsil were preceded by an identifiable cutaneous lesion, which contrasts with the current report. MCCUP presents a diagnostic dilemma that makes determining appropriate treatment regimens difficult. Further studies to distinguish whether these tumors originate from regressed cutaneous primaries or represent primaries themselves would be helpful for more accurate staging and clinical management.
